# Knocking Down Low Molecular Weight Protein Tyrosine Phosphatase (LMW-PTP) Reverts Chemoresistance through Inactivation of Src and Bcr-Abl Proteins

**DOI:** 10.1371/journal.pone.0044312

**Published:** 2012-09-05

**Authors:** Paula A. Ferreira, Roberta R. Ruela-de-Sousa, Karla C. S. Queiroz, Ana Carolina S. Souza, Renato Milani, Ronaldo Aloise Pilli, Maikel P. Peppelenbosch, Jeroen den Hertog, Carmen V. Ferreira

**Affiliations:** 1 Laboratory of Bioassays and Signal Transduction, Biochemistry Department, Biology Institute, University of Campinas (UNICAMP), Campinas, São Paulo, Brazil; 2 Center for Experimental Molecular and Medicine, Academic Medical Center, Amsterdam, The Netherlands; 3 Department of Gastroenterology and Hepatology, Erasmus MC-University Medical Center, Rotterdam, The Netherlands; 4 Universidade Federal do ABC, Santo André, São Paulo, Brazil; 5 Department of Organic Chemistry, Institute of Chemistry, University of Campinas (UNICAMP), Campinas, São Paulo, Brazil; 6 Hubrecht Institute, Utrecht, The Netherlands; Universidade Federal do Rio de Janeiro, Brazil

## Abstract

The development of multidrug resistance (MDR) limits the efficacy of continuous chemotherapeutic treatment in chronic myelogenous leukemia (CML). Low molecular weight protein tyrosine phosphatase (LMW-PTP) is up-regulated in several cancers and has been associated to poor prognosis. This prompted us to investigate the involvement of LMW-PTP in MDR. In this study, we investigated the role of LMW-PTP in a chemoresistant CML cell line, Lucena-1. Our results showed that LMW-PTP is highly expressed and 7-fold more active in Lucena-1 cells compared to K562 cells, the non-resistant cell line. Knocking down LMW-PTP in Lucena-1 cells reverted chemoresistance to vincristine and imatinib mesylate, followed by a decrease of Src and Bcr-Abl phosphorylation at the activating sites, inactivating both kinases. On the other hand, overexpression of LMW-PTP in K562 cells led to chemoresistance to vincristine. Our findings describe, for the first time, that LMW-PTP cooperates with MDR phenotype, at least in part, through maintaining Src and Bcr-Abl kinases in more active statuses. These findings suggest that inhibition of LMW-PTP may be a useful strategy for the development of therapies for multidrug resistant CML.

## Introduction

Failure of cancer chemotherapy may occur due to increased efflux of chemotherapeutic agents, leading to reduction of intracellular drug levels and consequent drug insensitivity, often to multiple agents. A well-established cause of multidrug resistance (MDR) involves increased expression of members of the ATP-binding cassette (ABC) transporter superfamily, which extrude various chemotherapeutic compounds from cells. In this context, resistance to chemotherapy is a major drawback in the effective treatment of chronic myelogenous leukemia (CML). Although initially most cases react favourably to chemotherapy, the build-up of resistance remains a major problem [1], [2]. Therefore, the identification of new processes involved in cancer resistance may open a new avenue to improve chemotherapy.

Low Molecular Weight Protein Tyrosine Phosphatases (LMW-PTP) are a family of enzymes related to various cellular events, such as cytoskeleton rearrangement, cell growth and modulation of immune response [3], [4]. Overexpression of LMW-PTP has been reported in several tumors and is generally associated with a proliferative phenotype and poor prognosis [5]. The LMW-PTP family has been reported to interact with several tyrosine kinases including PDGF [6], JAK2 [7], STAT5 [8], Insulin receptor, Eph Receptor [9] and EGF receptor [10]. However, the role of LMW-PTP in CML has not yet been well characterized.

In this study we report for the first time the contribution of LMW-PTP in the maintenance of chronic myeloid leukemia chemoresistance. We used the erythroleukemia cell line K562 and its resistant counterpart Lucena-1, which displays a high expression of the ABC-transporter P-glycoprotein (P-gp). We observed that LMW-PTP is overexpressed and highly active in Lucena-1 cells. Furthermore, overexpression of LMW-PTP in K562 cells impaired vincristine-induced cell death. On the other hand, knock down of LMW-PTP sensitizes Lucena-1 cells to vincristine (VCR), suggesting a causal role for LMW-PTP in resistance.

## Materials and Methods

### Cell line and Reagents

K562 cells were purchased from the American Type Culture Collection (ATCC, Rockville, MD) and the resistant cell line Lucena-1 was produced as described previously [11]. Anti-sheep, anti-rabbit, anti-goat and anti-mouse peroxidase-conjugated antibodies were purchased from Cell Signaling Technology (Beverly, MA). Antibodies against GAPDH and β-actin were purchased from Santa Cruz Biotechnology (Santa Cruz, CA). LMW-PTP (ACP1) antibody was obtained from Abcam. Src, p-Src, FAK, p-FAK and p-c-ABL antibodies were purchased from Cell Signaling. Monoclonal anti-P-glycoprotein (MDR) Clone F4, (Mouse Ascites Fluid, Product No. P 7965) was from Sigma. Caspase 3 kit assay from R & D Systems.

### Cell Culture

K562 and Lucena-1 cells were routinely grown in suspension in RPMI 1640 medium supplemented with 2 mM glutamine, 100 U/mL penicillin, 100 µg/ml streptomycin and 10% heat-inactivated fetal bovine serum, at 37°C in a 5% CO_2_ humidified atmosphere. VCR 60 nM was routinely added to Lucena-1 culture medium [11].

### Cell Viability

Cells were plated at 1×10^5^ cells/mL and treated with VCR or imatinib mesylate for 24 h. After treatment, cell viability was assessed by trypan blue dye exclusion [12].

### Phosphatase activity

To quantify the phosphatase activity, cells were lysed with 200 µL of Lysis Buffer (20 mM HEPES, pH 7,7 with 2,5 mM MgCl_2_, 0,1 mM EDTA, 20 mM p-nitrofphenilphosphate, 1 mM O-vanadate, 1 mM PMSF, 1 mM DTT, 10 µg/mL aprotinin e 10 µg/mL leupeptin) on ice for 2 h. After clarifying by centrifugation, the cell extract was incubated with antibodies against LMW-PTP overnight at 4°C under rotation. Later, A-Sepharose Protein was added to cell homogenate and incubated for 2 h at 4°C. The cell extract was washed 3 times with lysis buffer and 2 times with acetate buffer 100 mM pH5.5. The precipitate was ressuspended in acetate buffer 100 mM pH 5.5 and immediately was used for enzymatic assay.

PTP activity was measured using the Protein Tyrosine Phosphatase Assay kit Non Radioactive from Sigma (St. Louis, MO). Briefly, reaction medium contained 80 mM Acetate Buffer pH5.5, and 0.2 mM of PTP phosphorylated substrate. The reaction was carried at 37°C for 20 min and stopped with an equal volume of Malachite Green/Ammonium Molybdate reagent (1∶100). The amount of phosphate produced in the reaction was measured at 650 nm, in a microplate reader (ELx 800 BIO-TEK), and compared to a standard curve.

### Western Blotting Analysis

Cells (3×10^7^) were lysed in 200 µL of lysis buffer (50 mM Tris–HCl pH 7.4), 1% Tween 20, 0.25% sodium deoxycholate, 150 mM NaCl, 1 mM EGTA, 1 mM Na_3_VO_4_, 1 mM NaF and protease inhibitors (1 µg/ml aprotinin, 10 µg/ml leupeptin, and 1 mM 4-(2-aminoethyl) benzenesulfonyl-fluorid-hydrochloride)] for 2 h on ice. Protein extracts were cleared by centrifugation and protein concentrations were determined using the Lowry method. Twice the volume of SDS gel loading buffer (100 mM Tris–HCl pH 6.8), 200 mM DTT, 4% SDS, 0.1% bromophenol blue and 20% glycerol) was added to the samples, which were subsequently boiled for 5 min. Cell extracts were resolved by SDS-polyacrylamide gel (12%) electrophoresis (PAGE) and transferred to nitrocellulose membranes. Membranes were blocked in 5% fat-free dried milk or bovine serum albumin in Tris-buffered saline-Tween 20 (0.1%) and incubated overnight at 4°C with the appropriate primary antibody at 1∶1000 dilution. After washing in TBS-Tween 20 (0.1%), membranes were incubated with anti-rabbit, anti-mouse and anti-goat horseradish peroxidase-conjugated secondary antibodies, at 1∶5000 dilutions, in blocking buffer for 1 h. Proteins were detected using enhanced chemiluminescence.

### Transfection of K562 Cells with LMW-PTP Plasmid

K562 cells (100.000 cells/mL) were grown for 24 h and the transfections were done using the Effectene transfection kit (QIAGEN Benelux, Venlo, Netherlands) according to the manufacturer’s instructions. Briefly, the cells were transfected with 2.4 µg of pcDNA3.1/V5-His-TOPO vector with or without an insert containing the sequence of human LMW-PTP after a cytomegalovirus promoter. Overexpression was always verified by Western blotting.

### Transfection of K562 and Lucena-1 Cells with LMW-PTP siRNA

Lucena-1 cells (100.000 cells/mL) were grown for 24 h and subsequently transiently transfected with LMW-PTP siRNA (Qiagen cat.# SI02776851). Transfections were done using the Hiperfect transfection kit (QIAGEN Benelux, Venlo, Netherlands) according to the manufacturer’s instructions. Briefly, the cells were transfected with LMW-PTP siRNA (final concentration: 5 nM) for 72 h, and then washed with Phosphate Buffer Saline and lysed with a specific buffer for Western blotting procedure. Lysates were subsequently mixed with loading buffer for electrophoresis. The efficiency of transfection was assessed based on the expression of LMW-PTP by Western blotting analysis.

### Caspase 3 Activity Assay

After silencing procedure the Lucena-1 was treated with VCR or imatinib for 24 h. Afterwards, the cells were centrifuged and washed with PBS. Caspase activity was determined by the measurement at 405 nm of *p*-nitroaniline released from the cleavage by caspase 3 (Ac-DEVD-*p*NA) substrates, according to manucfaturers instructions (R & D Systems). The enzyme activity was expressed in pmol/mL/min and the extinction coefficient of *p*NA was 10,000 M^−1^ cm^−1^.

### P-Glycoprotein Activity Evaluation

To measure P-gp activity we used Rhodamine 123, one of its substrates. After silencing LMW-PTP, cells were incubated with 200 ng/mL Rhodamine 123 for 30 minutes with or without a P-gp inhibitor (5 µM verapamil). Afterwards, cells were washed with Phosphate Buffer Saline and incubated in culture media with or without P-gp inhibitor for 1 h at 37°C, washed and suspended in cold PBS and kept on ice. Rhodamine fluorescence was subsequently assessed by flow cytometry analysis within the live-gate, using a FACScalibur (Becton and Dickinson, USA). Data were analyzed in the WinList software and the mean fluorescence intensity was plotted in the graph. The P-gp activity is correlated to Rhodamine-123 fluorescence intensity [11].

### Statistical Analysis

All experiments were performed in triplicate and results were shown in the graphs as means ± S.E.M. Soluble lysates were matched for protein content and analyzed by Western blotting. Analysis of immunoblotting band density was performed by Scion Image® software (Frederick, MD, USA). All bands were compared with their respective internal control. Differences were analyzed using a one-way analysis of variance (ANOVA) followed by Bonferoni posttest. p values < 0.05 were considered significant. All data were analyzed using GraphPad Prism Software, Version 5.0.

## Results

### LMW-PTP is Highly Expressed and Active in Resistant Leukemia Cells

**Figure 1:**
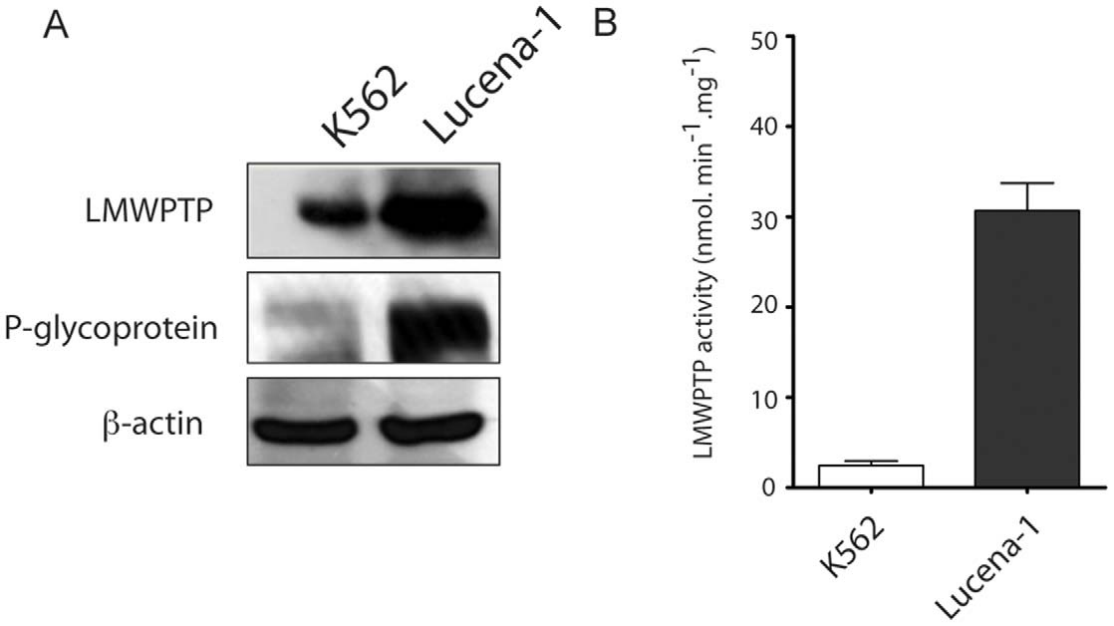
P-glycoprotein and LMW-PTP on both leukemia cell lines. Cells (100.000/ mL) were plated and lysed after 24 h. (**A**) The expression of P-gp and LMW-PTP were evaluated by immunoblotting. Soluble lysates were matched for protein content and analyzed by Western blot and β-actin was used as an internal control. (**B**) LMW-PTP from K562 and Lucena-1 cells was immunoprecipitated and afterwards had its activity determined by using p-nitrophenyl phosphate as a substrate.

Initially, we verified the leukemia resistance model by checking P-gp expression in K562 and its resistant counterpart Lucena-1 cells. As shown in Figure 1A, Lucena-1 cells highly express P-gp, indicating that this cell line is an adequate model to study intracellular resistance mediators that might contribute to maintaining the resistant phenotype.

Subsequently, we analyzed the expression levels of LMW-PTP and its activity in both cell lines, K562 and Lucena-1. Figure 1A shows the overexpression of LMW-PTP in Lucena-1 cells when compared to K562 cells. Figure 1B shows that Lucena-1 cells present high LMW-PTP activity, about 7-fold higher than in K562.

### Overexpression of LMW-PTP in K562 Cells Decreases Sensitivity towards Vincristine

**Figure 2:**
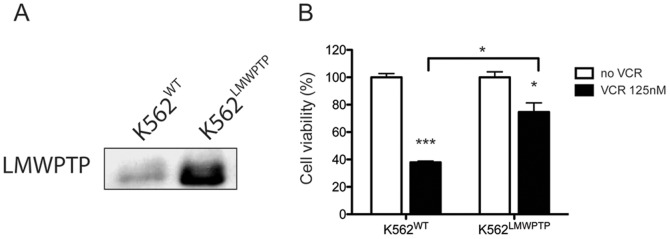
K562 cells overexpressing LMW-PTP are less sensitive towards vincristine. (A) LMW-PTP was overexpressed in K562 and its expression was examined in the total lysate of leukemia cells. (**B**) K562 cells overexpression LMW-PTP (K562^LMW-PTP^) or not (K562^WT^) were treated with VCR 125 nM for 24 h and the viable cells were counted. Each value represents the mean ± S.D. of three independent experiments (n = 3). ***p<0.001, *p<0.5 - significant differences relative to control (no VCR) or as indicated in the graph.

In order to provide more information about the possible involvement of LMW-PTP on resistant phenotype maintenance, we overexpressed LMW-PTP through transient transfection in K562 cells (Figure 2A) and subsequently treated these cells with VCR. Interestingly, VCR reduced cell viability of K562 wild type cells (K562^WT^) to 37,8% (±1,7) while in K562 overexpressed LMW-PTP (K562^LMW-PTP^) treated with VCR the cell viability was 74,6% (±11,6) compared to their respective non-treated cells (Figure 2). Thus, it is clear that overexpression of LMW-PTP protected the K562 cells against cell death induced by VCR. These results strongly suggest that LMW-PTP is involved in chemoresistance and its overexpression makes the cells more resistant.

### *LMW-PTP* Silencing Increases Lucena-1 Cells Sensitivity towards Vincristine and Imatinib

**Figure 3:**
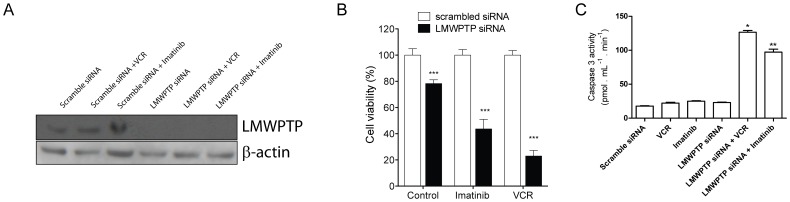
*LMW-PTP* silencing increases the cytotoxicity effect of chemotherapeutics on Lucena-1 cells. (**A**) LWM-PTP was knocked down in Lucena-1 cells and its expression was checked by Western (**B**) After 48 h of *LMW-PTP* silencing Lucena-1 cells (100.000 cells/ml) were treated with VCR 500 nM or imatinib 500 nM for 24 h, subsequently, the viable cells were assessed by trypan blue exclusion and (**C**) apoptosis induction by caspase 3 activity. Each value represents the mean ± S.E.M. of three independent experiments (n = 3) and has been normalized to scramble siRNA sample. *** p<0.001 - significant differences relative to its respective scramble siRNA sample.

To confirm the role of LMW-PTP in chemoresistance, Lucena-1 cells were treated with VCR or imatinib mesylate (Gleevec®) for 24 h after *LMW-PTP* silencing. Figure 3A shows the efficiency of knocking down LMW-PTP in Lucena-1 cells. Figure 3B shows that Lucena-1 cells become more sensitive to both drugs when *LMW-PTP* is knocked down. In this experiment we set the scramble siRNA transfected cells as 100% of viability, to allow comparison to LMW-PTP siRNA transfected cells. Only the transfection of *LMW-PTP* siRNA in Lucena-1 cells displayed a minor effect of decreasing cell viability to approximately 80%. Imatinib 500 nM decreased cell viability to 43.6 (±7.3) in *LMW-PTP* knocked down Lucena-1 cells. VCR 500 nM decreased the number of viable cells to 22.9% (±4.2). In addition, the induction of apoptosis was also addressed by examining the activity of caspase 3. As shown in Figure 3, an expressive increase of caspase 3 activity was observed in silenced Lucena-1 cells treated with imatinib or VCR. It is important to mention that VCR and imatinib displayed a slight effect on Lucena-1 wild type viability, reduction of only 10% and 30% of cell viability, respectively (data not shown). These results emphasize the role of LMW-PTP in CML chemotherapy resistance.

### LMW-PTP does not Affect P-gp Activity

**Figure 4:**
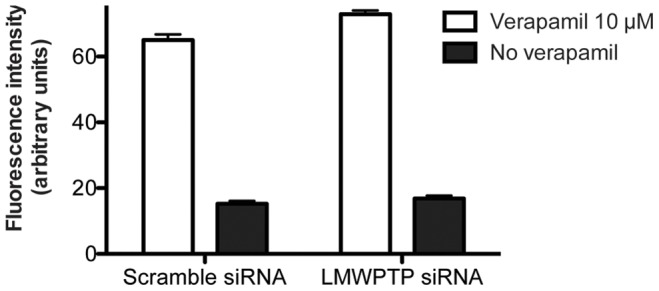
Evaluation of P-glycoprotein activity. To measure P-gp activity the substrate Rhodamine 123 was used as substrate. *LMW-PTP* was silenced and after 24 h cells were further incubated for 1 h in the presence of 200 ng/mL Rhodamine 123 with or without a P-gp inhibitor (5 µM verapamil). Afterwards, cells were washed with PBS and Rhodamine 123 mean fluorescence intensity was assessed by flow cytometry analysis within the live-gate, using a FACScalibur (Becton and Dickinson, USA). Decreasing of mean fluorescence intensity indicates higher P-gp activity.

Since LMW-PTP silencing in Lucena-1 cells made them more sensitive to chemotherapeutic, we hypothesized that this phosphatase can interfere with P-gp activity. In Figure 4 we showed the mean fluorescence intensity of Rhodamine 123 in live-gated Lucena-1 cells after LMW-PTP silencing. The P-gp activity is correlated with Rhodamine 123 fluorescence intensity: more fluorescence intensity indicates less P-gp activity, while lower fluorescence intensity indicates higher P-gp activity. In Figure 4 we observed that the mean fluorescence intensity in LMW-PTP knocked down Lucena-1 cells had no changes compared to scramble siRNA transfected cells. As a positive control, we used verapamil 5 µM, an inhibitor of P-gp. Thus, silencing LMW-PTP presented no change in P-gp function. This result suggests that the role of LMW-PTP in chemoresistance is related to another molecular mechanism instead of directly regulating P-gp activity.

### LMW-PTP is Upregulated by Vincristine Treatment in Lucena-1 Cells

**Figure 5:**
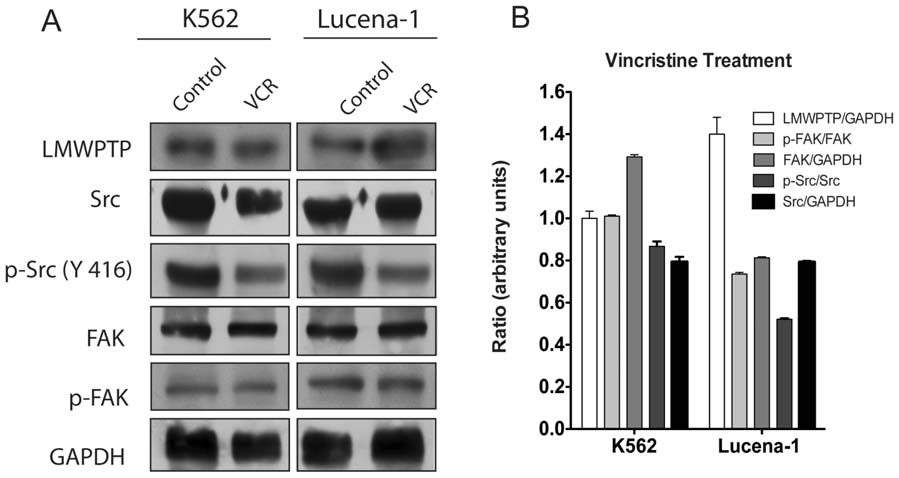
Expression of LMW-PTP and modulation of c-Src and FAK in leukemia cells treated with vincristine. Cells were treated with VCR for 24 h and LMW-PTP expression, phosphorylation status of c-Src and FAK were analyzed. (A) K562 and Lucena-1 cells were treated with VCR for 24 h and the protein levels were analyzed by Western blotting. (B) The ratio between LMW-PTP/GAPDH, Src/GAPDH, FAK/GAPDH, p-Src/Src, p-FAK/FAK from VCR treatment samples were quantified by densitometry and plot in the graph.

To get more evidence about the role of LMW-PTP in chemotherapy resistance, we checked the expression of LMW-PTP, Src and FAK in K562 and Lucena-1 cell lines treated with VCR. Interestingly, VCR induced high expression of LMW-PTP in Lucena-1 cells but not in K562 (Figures 5A and 5B). Src expression and phosphorylation at Y416, its stimulatory residue, were downregulated by VCR treatment in both leukemic cells. Importantly, the phosphorylation (Y576 and Y577) levels of FAK remained unchanged in K562 cells, while a decrease was observed in Lucena-1 cells (Figure 5B). The total levels of Src decreased by VCR treatment in both cell lines, but FAK had a slight increase in K562 cells. These results indicate that VCR treatment interferes with Src activity.

### Knocking Down LMW-PTP Decreases Phosphorylation of Src and Abl

**Figure 6:**
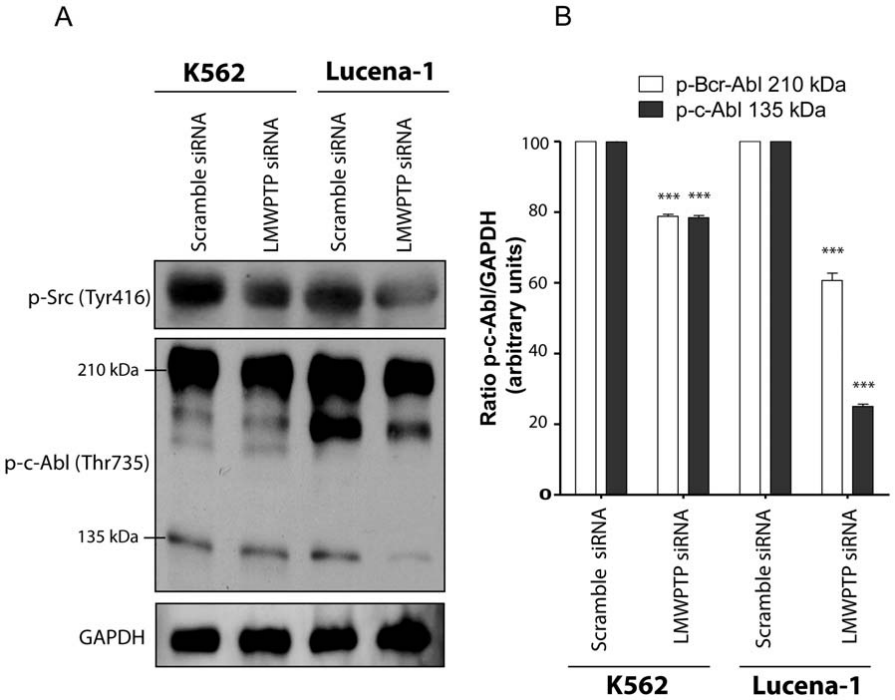
Knocking down *LMW-PTP* decreased phosphorylation of Src and ABL. (**A**) Phosphorylation status of Src kinase and ABL in leukemia cells with silenced *LMW-PTP*. (**B**) The ratio between p-Brc-Abl(210 kDa)/GAPDH and p-c-Abl (135 kDa)/GAPDH, from K562 and Lucena-1 cells with silenced *LMW-PTP* (*LMW-PTP* siRNA) or not (scramble siRNA) were quantified by densitometry and plot in the graph. *** p<0.001- significant differences relative to the respective scramble siRNA.

To further investigate the molecular mechanism by which LMW-PTP increases chemoresistance in CML, we analyzed the levels of phospho-Src and phospho-ABL after knocking down LMW-PTP in K562 and Lucena-1 cells. In Figure 6A we observed that phosphorylation of Src at Tyr 416 was decreased in K562 and Lucena-1 cells after LMW-PTP knockdown. The same was observed for p-Bcr-ABL and p-c-Abl in Figure 6A: phosphorylation at Thr 735 residue from single c-Abl (135 kDa) and Brc-Abl (210 kDa) was decreased by LMW-PTP silencing in both cell lines. Figure 6B shows the densitometry analysis of the bands (p-c-Abl and p-Bcr-Abl) obtained in Western blotting assay, normalized by GAPDH.

## Discussion

Improving chemotherapy for neoplastic diseases constitutes one of the most important challenges in biomedical research. Among the major issues when dealing with leukemic disease is the build-up of resistance against therapy [13], [14]. This may include resistance associated with decreased drug accumulation in the cell, altered intracellular drug distribution, increased detoxification, diminished drug-target interaction, increased DNA repair, altered cell-cycle regulation and uncoupling of pathways linking cellular damage with programmed cell death [13], [14]. Suppression of tumorigenesis often involves modulation of signal transduction pathways, leading to modulation of gene expression, cell cycle progression or apoptosis.

This study shows for the first time that LMW-PTP is involved in chemoresistance. Firstly, the resistant cell line, Lucena-1, presents high activity and expression of LMW-PTP compared to the non-resistant cells, K562. Secondly, LMW-PTP overexpression leads to resistant phenotype in non-resistant cells and its silencing induces chemotherapy sensitivity in resistant cells, as clearly indicated by apoptosis induction in those cells. LMW-PTP has already been recognized as a possible positive mediator in tumor onset and progression. In different tumor types the overexpression of this phosphatase is generally a prognosis for more aggressive cancer development [15], [5], [4]. Thus, this study contributes to elucidate one more role of LMW-PTP in tumor progression.

We hypothesized that LMW-PTP can act directly on P-gp. However, it was refuted because knocking down LMW-PTP in Lucena-1 cells did not change P-gp function. This result led us to focus on the molecular mechanism of chemoresistance. Protein phosphorylation is a post-translational modification that can inhibit or activate a particular protein. Since cancer cells usually present upregulation of kinases, such as the non-receptor tyrosine kinases, focal adhesion kinase FAK [16] and Src [17], and these kinases are regulated by multiple phosphorylation events, we focused our study on those kinases. Importantly, our findings suggest that LMW-PTP contributes for Lucena-1 resistance by maintaining the activation of Src and Bcr-Abl.

VCR treatment leads to overexpression of LMW-PTP in Lucena -1 cells, suggesting a cellular response to counterbalance the death-inducing stimuli. On the other hand, VCR downregulates FAK and Src. FAK is located in focal adhesions and coordinates signals from integrins, cytokines, growth factor receptors and oncogenes, playing an important role in cell motility and survival through extracellular signal-regulated kinase (ERK), PI3K/Akt, MAPK and JAK/STAT signaling pathways [18]. FAK has been shown to be elevated in a variety of cancers and frequently correlates to poor patient prognosis [16]. Besides, modulation of FAK expression and (or) phosphorylation influences the sensitivity of tumor cells to diverse chemotherapeutic agents [16].

Silencing *LMW-PTP* in Lucena-1 cells downregulated Src (Figure 6). Src kinase family proteins are expressed in hematopoietic cells and are involved in signaling pathways that regulate cell growth and proliferation [19], [20]. Src can be activated by cytoplasmic proteins such as, FAK or its molecular partner Crk-associated substrate (CAS). Elevated Src activity may be caused by increased expression or by deregulation due to overexpression of its upstream mediators such as FAK, PDGFR, FGFR, integrin and VEGFR [21]. In multiple tumors, overexpression and activation of both c-Src and FAK have been demonstrated to lead to increased invasive and metastatic potential [22]. Houanwen and coauthors (2009) showed that nilotinib-resistant K562 cells present up-regulation of the Src family kinase, p53/56 Lyn, and silencing of p53/56 Lyn by siRNA in nilotinib-resistant K562 cells restored their sensitivity to nilotinib [16].

CML cells, such as K562 and Lucena-1, are characterized by the presence of the Philadelphia chromosome translocation that contains the BCR-ABL hybrid gene, which encodes an oncogenic fusion protein Bcr-Abl. This fusion protein displays deregulated protein tyrosine kinase activity that is responsible for leukemogenesis in vitro and in vivo [23]. For this reason, Bcr-Abl has been considered as a target for the therapy of CML and BCR-ABL-positive ALL [17]. In this study we demonstrated that *LMW-PTP* knockdown in CML cells decreased Abl phosphorylation levels at its activating site. This finding brings more evidence to our hypothesis that this phosphatase might contribute for both CML cell survival and resistance.

In conclusion, this study contributes to elucidate the role of LMW-PTP in leukemia aggressiveness, showing for the first time that LMW-PTP is involved in resistance, through maintaining both Src and Bcr-Abl kinases in a more active status. However, this mechanism is not completely elucidated and needs further studies.
